# Comparative Functional Analysis of *ZFP36* Genes during *Xenopus* Development

**DOI:** 10.1371/journal.pone.0054550

**Published:** 2013-01-16

**Authors:** Karine Tréguer, Corinne Faucheux, Philippe Veschambre, Sandrine Fédou, Nadine Thézé, Pierre Thiébaud

**Affiliations:** 1 Université de Bordeaux, Bordeaux, France; 2 CNRS UMR 5164, Bordeaux, France; University of Colorado, Boulder, United States of America

## Abstract

ZFP36 constitutes a small family of RNA binding proteins (formerly known as the TIS11 family) that target mRNA and promote their degradation. In mammals, ZFP36 proteins are encoded by four genes and, although they show similar activities in a cellular RNA destabilization assay, there is still a limited knowledge of their mRNA targets and it is not known whether or not they have redundant functions. In the present work, we have used the *Xenopus* embryo, a model system allowing gain- and loss-of-function studies, to investigate, whether individual ZFP36 proteins had distinct or redundant functions. We show that overexpression of individual amphibian zfp36 proteins leads to embryos having the same defects, with alteration in somites segmentation and pronephros formation. In these embryos, members of the Notch signalling pathway such as *hairy2a* or *esr5* mRNA are down-regulated, suggesting common targets for the different proteins. We also show that mouse Zfp36 protein overexpression gives the same phenotype, indicating an evolutionary conserved property among ZFP36 vertebrate proteins. Morpholino oligonucleotide-induced loss-of-function leads to defects in pronephros formation, reduction in tubule size and duct coiling alterations for both *zfp36* and *zfp36l1*, indicating no functional redundancy between these two genes. Given the conservation in gene structure and function between the amphibian and mammalian proteins and the conserved mechanisms for pronephros development, our study highlights a potential and hitherto unreported role of *ZFP36* gene in kidney morphogenesis.

## Introduction

Zinc-finger-containing-proteins constitute the most abundant protein superfamily in eukaryote genomes and they are involved in various cellular processes through their binding to DNA, RNA or protein [1]. Among this super family are subfamilies of proteins containing a variable number of zinc finger motifs based on a cysteine-histidine repeat with the configuration cys-cys-cys-his (C3H) [2]. One subclass of this family contains proteins that possess two C3H type zinc finger domains Cx_8_Cx_5_Cx_3_H (where x is a variable amino acid) or a Tandem Zinc Finger domain (TZF) separated by an 18 amino acids linker region. The prototype of this family is named ZFP36, previously described as TIS11, Tristetraprolin (TTP), Nup475 and GOS24 and which is rapidly induced by several mitogens [3], [4], [5], [6], [7]. Depending on the species, two or three other *ZFP36* genes have been found in vertebrates. In human, in addition to *ZFP36*, there are two other genes namely *ZFP36L1* and *ZFP36L2*. In rodents a fourth gene, *Zfp36L3*, has been identified and shown to be expressed only in placenta [8]. ZFP36 proteins have been showed to bind to AU rich elements (ARE) present in the 3′UTR region of several mRNA encoding cytokines like Tumor Necrosis Factor alpha (TNFα) or the Granulocyte-Macrophage Colony-Stimulating Factor (GM-CSF) and this binding involves the tandem zinc finger domain of the proteins [9], [10], [11], [12], [13]. As a consequence, mice deficient for *Zfp36* by gene targeting although appear normal at birth, soon develop a complex syndrome related to medullar and extramedullar myeloid hyperplasia associated with an increased cellular concentration of *TNFα* mRNA [14]. Inactivation of *Zfp36l1* gene in mouse by knockout leads to the death of the embryo *in utero* at about 11 days of development by failure of chorioallantoic fusion, the embryos showing extraembryonic and intraembryonic vascular abnormalities along with heart defects [15], [16]. Mutation of *Zfp36l2* gene in the mouse causes female infertility and together, these knockout studies suggest distinct and non redundant functions for *ZFP36* genes during development [17]. Mice lacking *Zfp36l1* and *Zfp36l2* genes during thymus development are prone to acute lymphoblastic leukemia and show elevated *Notch1* mRNA levels [18], illustrating the importance of those RNA binding proteins during organ development and homeostasis.

Members of the *ZFP36* gene family have been identified in several metazoans such as *Drosophila*, zebrafish and more recently in mollusc [19], [20], [21], [22], [23]. In the amphibian *Xenopus laevis*, four distinct genes that code proteins containing a tandem zinc finger domain have been identified and named *xC3H-1* to *4* [24], [25]. X*C3H-1, xC3H-2* and *xC3H-3* genes are true orthologs of the human *ZFP36, ZFP36L1* and *ZFP36L2* genes respectively. *xC3H-4* is distinct from other *ZFP36* genes, being unique to amphibians and encoding a protein with two tandem zinc fingers instead of one [24], [25]. In agreement with *Xenopus* Gene Name guidelines, we will refer *xC3H-4* to *zfp36l4* and use *zfp36*, *zfp36l1* and *zfp36l2* for the other members of the family. *Zfp36l1* and *zfp36l2* have been showed, either by gain-of-function (for *zfp36l1*) or by gain and loss-of-function (for *zfp36l2*) to be involved in *Xenopus* pronephros formation while *zfp36l4* has been shown to regulate meiosis [25], [26], [27]. However, no functional study has been performed yet on *zfp36*, the prototype of the family and no comparative functional study between the different zfp36 proteins has been undertaken.

To gain more insight into the evolutionary history of *ZFP36* genes, we have compared in detail their genomic structure between various metazoan phyla and found that vertebrates and basal metazoan *ZFP36* genes are structurally conserved while protostome genes have diverged. In order to complete our knowledge on the amphibian *zfp36* gene family, we have analyzed the developmental expression of *zfp36* gene and performed a functional analysis. We found that the amphibian *zfp36* gene has a unique expression pattern during development, one that is associated with somitic segmentation and nephrogenesis. When overexpressed in *Xenopus* embryos, each member of the *zfp36* gene family gives the same embryonic defects suggesting common targets to all members of the family. We have identified several mRNAs whose expression is abolished or strongly reduced when the different *zfp36* mRNA are overexpressed and in morphant embryos. Because zfp36 proteins are potential regulator of mRNA deadenylation and translation we may hypothesize they act on those mRNAs to regulate an early phase of organogenesis.

## Results

### The structural organization of *ZFP36* genes is conserved between evolutionary distantly related animals

**Figure 1:**
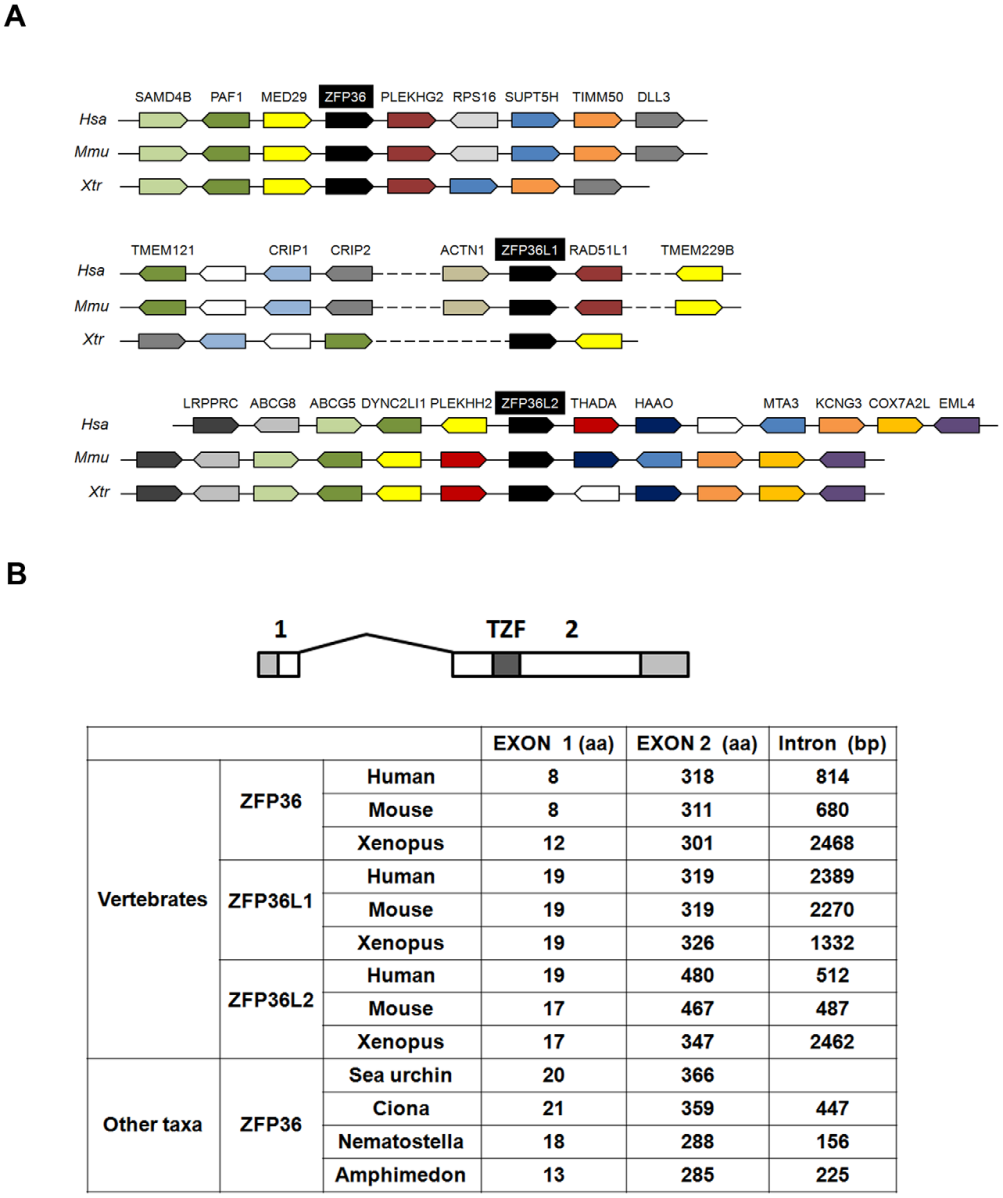
Conservative evolution of vertebrate *zfp36* genes. (A) Conserved syntenic regions between human (*Hsa*), mouse (*Mmu*) and *Xenopus tropicalis* (*Xtr*) chromosome regions containing *zfp36*, *zfp36l1* and *zfp36l2*. Gene names symbols are according to HUGO. Boxes with the same colour correspond to the same gene; white boxes correspond to genes without annotation or without orthologues in the species shown here. The drawing is not to scale to avoid complexity and dashes represent long chromosome regions. (B) Conserved structural organization of vertebrates *zfp36* genes between evolutionary distant animals. Exons (1, 2) are figured in open boxes and intron as a solid line respectively. Shaded box, untranslated region. TZF, Tandem Zing Finger domain.

Genes encoding proteins containing two C3H type zinc finger domains (Cx_8_Cx_5_Cx_3_H) (or TZF for Tandem Zinc Finger) have been independently cloned by several groups and identified by a variety of names (see introduction). In accordance with recommendations of the HUGO Gene Nomenclature Committee (http://www.genenames.org/), we propose to use ZFP36 as the founder name for members of this family, in place of the previous designations Tis11 or TTP. Therefore, in addition to the three human genes *ZFP36, ZFP36L1* and *ZFP36L2*, the fourth gene identified in rodents and belonging to this family is named *Zfp36l3*. Among the four genes containing a TZF domain described in *Xenopus, xC3H-1, xC3H-2* and *xC3H-3* are true orthologs of the human *ZFP36, ZFP36L1* and *ZFP36L2* as confirmed by synteny and phylogenetic analyses (Fig. 1A and Fig. S1). The fourth *Xenopus zfp36* gene, *xC3H-4,* is distinct from other *ZFP36* genes and unique to amphibian genome [24], [25]. In agreement with *Xenopus* genes names guidelines, we will refer *xC3H-4* to *zfp36l4* and use *zfp36, zfp36l1* and *zfp36l2* for the other members of the family.

*ZFP36* genes have been identified in numerous metazoans but no comparative analysis based on gene structure has been performed yet on those genes. In order to obtain a more comprehensive picture of the *ZFP36* gene family in metazoan, we searched in EST and genomic databases for the presence of genes containing a conserved TZF domain in several metazoan taxa. We identified a unique gene containing a TZF domain in the sea anemone *Nematostella vectensis* that belongs to the basal metazoan cnidarians. A single gene coding a TZF containing domain was also identified in the genome of the sponge *Amphimedon queenslandica*, a member of an ancient group of animals that has diverged from other animals over 600 Ma. Similarly we found a single *ZFP36* gene in two basal deuterostomes bilateria, the echinoderm sea urchin *Strongylocentrotus purpuratus* and the urochordate *Ciona intestinalis.* A phylogenetic tree made with the TZF domain encoded by these different genes indicates that *ZFP36*, *ZFP36L1* and *ZFP36L2* have evolved from a single gene and that *ZFP36L1* and *ZFP36L2* are closely related and probably resulted from a duplication event during evolution (Fig. S1).

In order to gain insights into the evolution of the *zfp36* genes, we retrieved their genomic organization for different taxa and compared with with the vertebrate *ZFP36, ZFP36L1 and ZFP36L2* genes. The human, mouse and amphibian genes showed a highly conserved organization with two exons separated by a phase 0 intron of variable size (Fig. 1B). When compared to the situation in other taxa, one striking finding is that the gene structure observed in vertebrates extends not only to other deuterostomes (sea urchin and *ciona*) but also to basal metazoans such as cnidarians (*Nematostella*) and sponges (*Amphimedon*). In each case, the two exons are separated by a phase 0 intron and the TZF domain is always found in the second exon (Fig. 1B).

*Zfp36l3* and *zfp36l4* have a different gene structure from other *Zfp36* genes (data not shown). In rodents, *Zfp36l3* is intronless, suggesting that the gene has arisen in that lineage by retrotransposition of a processed cDNA (data not shown). In *Xenopus, zfp36l4* gene is constituted by two exons, but exon 1 contains only 5′ untranslated sequence (data not shown). This feature is also compatible with a retrotransposition event that may have occurred in the amphibian lineage.

In contrast to other taxa analysed, *zfp36* gene structure differs in protostomes. The unique *Drosophila zfp36* gene contains three exons separated by a phase 0 intron and a phase 2 intron respectively (data not shown) and the nematode *Caenorhabditis elegans zfp36* (ccch-1) comprises 9 exons (data not shown). Since the unique intron in the vertebrate *ZFP36, ZFP36L1 and ZFP36L2* genes is found at the same position and also in the basal orthologous genes of cnidarians and sponges, we conclude that this splice structure is an ancestral trait. The more complex gene structure observed in *Drosophila* and *C. elegans zfp36* genes reflects secondary lineage-specific gain of introns. Together this analysis reveals a strong conservation in the structure of the *ZFP36* gene in the deuterostome lineage. The three vertebrates genes *ZFP36, ZFP36L1* and *ZFP36L2* share the same structural organization and this is compatible with the gene duplication events that have occurred during vertebrates evolution [28].

### Comparative analysis of *zfp36* genes expression pattern during *Xenopus* development

**Figure 2:**
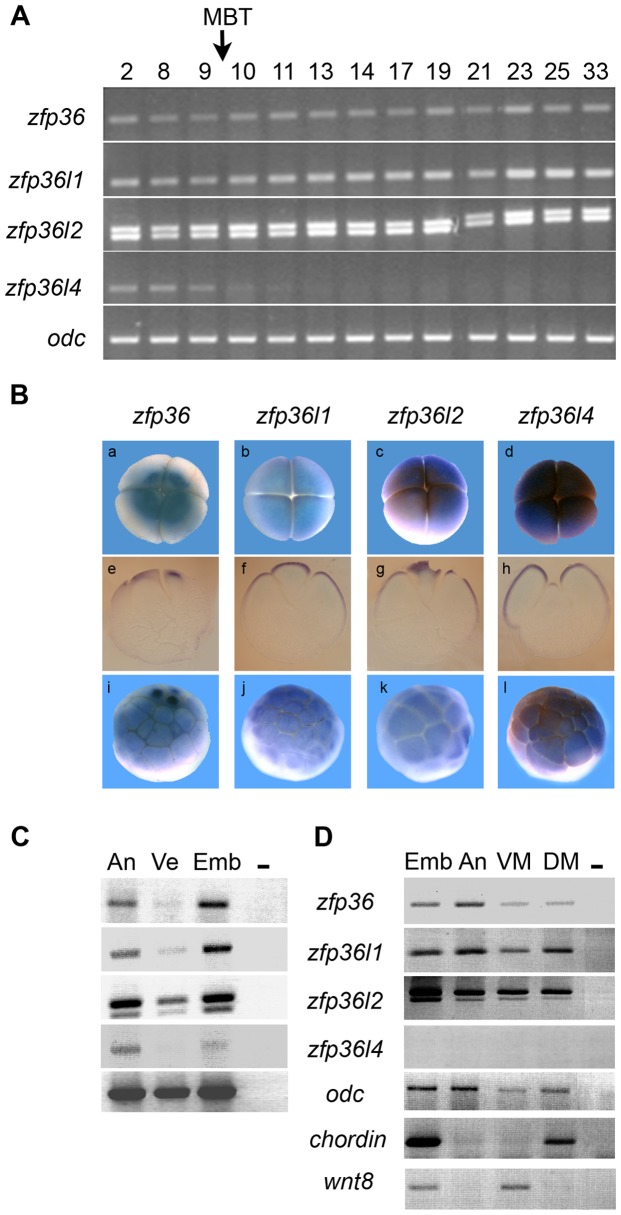
mRNA expression of *Xenopus zfp36* genes during development. (A) RT-PCR analyses showed that all *zfp36* genes are maternally expressed. *zfp36, zfp36l1* and *zfp36l2* mRNAs are expressed at a constant level throughout development from stage 2 to stage 33 while *zfp36l4* mRNA level decreases after the mid-blastula transition (MBT, arrow). (B) *In situ* hybridization showed that all four *zfp36* mRNA are localized at the animal pole in 4-cell stage (a–d) and morula stage (i–l) embyos. e–h correspond to histological sections from embryos shown in a–d. (C) RT-PCR analysis showed that *zfp36* mRNAs are preferentially expressed in the animal pole region of blastula embryos. (D) RT-PCR analysis showed that *zfp36* mRNAs are expressed throughout the embryo at the gastrula stage. An, animal pole; DM, dorsal marginal zone; Emb, whole embryo; Ve, vegetal pole; VM, ventral marginal zone. A control embryo (Emb) assayed by RT-PCR for the expression of control genes *chordin* and *wnt8*. *odc* was used as control of loading and a reaction was performed in the absence of reverse transcriptase to check for genomic DNA contamination (-).

The cloning and expression of members of the Tis11/TTP gene family in *Xenopus* has been previously reported [24], [25], [27], however these studies were primarily focused on *zfp36l1* or *zfp36l2* but not on *zfp36 (Tis11/TTP)* the founding member of the family. Moreover, no gene function study has been yet performed on *zfp36*. Before undertaking functional studies, we further evaluated *zfp36* expression during development and performed a detailed expression of *zfp36* as compared to other gene family members. RT-PCR analysis indicates that *zfp36l, zfp36l1* and *zfp36l2* genes are expressed maternally and throughout development at a constant level from egg to tadpole stage (Fig. 2A). In contrast, there is a decrease of *zfp36l4* mRNA level after fertilization, with complete disappearance after midblastula transition (Fig. 2A). These data confirm previous observations obtained by Northern blot for the four genes and by RT-PCR for *zfp36l2* [24], [25]. Because the four *zfp36* genes are maternally expressed, we analyzed the localization of their corresponding mRNAs. *In situ* hybridization combined with histological sections revealed that the four genes are expressed at the animal pole in 4-cell stage embryo (Fig. 2B, a–h). In the morula embryo, *zfp36* mRNA is concentrated at the animal pole while the three other mRNAs spread from the animal pole to the marginal zone (Fig. 2B, i–l). mRNA distribution was then analyzed at blastula stage by RT-PCR on dissected embryos. When the blastula embryo is dissected into animal versus vegetal pole, *zfp36, zfp36l1* and *zfp36l4* mRNAs are mostly found in the animal pole region while *zfp36l2* mRNA is also expressed in the vegetal pole (Fig. 2C). At the gastrula stage, after zygotic transcription had resumed, *zfp36, zfp36l1* and *zfp36l2* mRNAs are found in the animal pole, but also in ventral and dorsal mesoderm, indicating ubiquitous expression (Fig. 2D). As expected, *zfp36l4* is not expressed in gastrula stage embryo (Fig. 2D).

**Figure 3:**
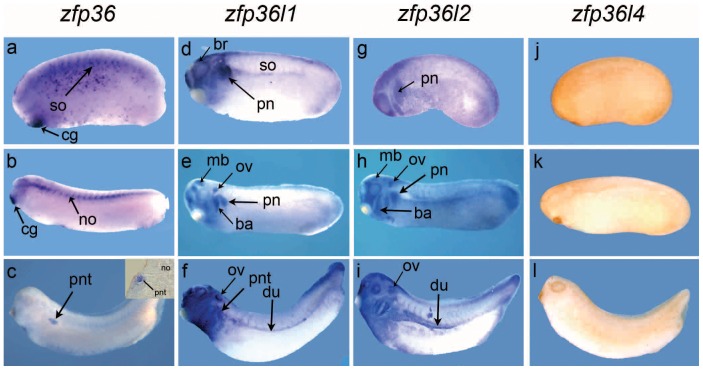
*Zfp36* has a distinct spatial expression from *zfp36l1* and *zfp36l2* in the embryo. The localization of *zfp36* mRNAs was detected by *in situ* hybridization in embryos from stage 24 (a, d, g, j), stage 28 (b, e, h, k) and stage 35/36 (c, f, i, l). A close up view of a transverse section at the level of pronephros is shown in c. *Zfp36l4* expression was never detected in the embryos at any stage. ba, branchial arches; br, brain; cg, cement gland; du, pronephric duct; mb, midbrain; no, notochord, ov; otic vesicle; pn, pronephros anlage, pronephric tubule; so, somites.

The spatial expression of the four *zfp36* genes was then analyzed on later stages embryo by whole mount *in situ* hybridization. *Zfp36* mRNA is detected in the somites, cement gland and appears as punctate staining in the lateral mesoderm of the stage 24 embryo (Fig. 3a). By stage 28, *zfp36* expression is restricted to the cement gland and appears in the notochord where it extends to its rostral end (Fig. 3b). In the stage 33/34 embryo, expression persists in the cement gland but notochord expression is no longer detected while the pronephric tubule is stained as seen in a close view section (Fig. 3c). *Zfp36l1* is mainly expressed in pronephros anlagen and brain of stage 24 embryos and at a lower level in somites (Fig. 3d). By stage 28 embryo, pronephros expression persists and additional sites of expression are detected in the midbrain, otic vesicle and branchial arches (Fig. 3e). At stage 35/36, the pronephros tubule and duct are both stained (Fig. 3f) as previously reported [27]. *Zfp36l2* expression is detected in pronephros anlagen in the stage 24 embryo where it persists through stage 28, with additional expression in branchial arches, otic vesicle and midbrain (Fig. 3g, h). By stage 35/36, *Zfp36l2* expression is detected in the pronephric duct and surrounds the pronephric tubule (Fig. 3i) as previously reported [25]. *Zfp36l4* mRNA is not detected in stage 24 nor at later stages (Fig. 3j–l) thus confirming our RT-PCR data (Fig. 2A) and previous Northern blot analysis [24].

Taken together, these results indicate that *zfp36* has a unique expression pattern when compared to other *zfp36* gene family members. Although *zfp36* is expressed during pronephros development like *zfp36l1* and *zfp36l2*, it is a late marker compared to the two other genes and its expression is restricted to the pronephric tubule whilst *zfp36l1 and zfp36l2* also mark the pronephric duct.

### Different effects of signalling pathways on *zfp36* expression in embryonic cells

**Figure 4:**
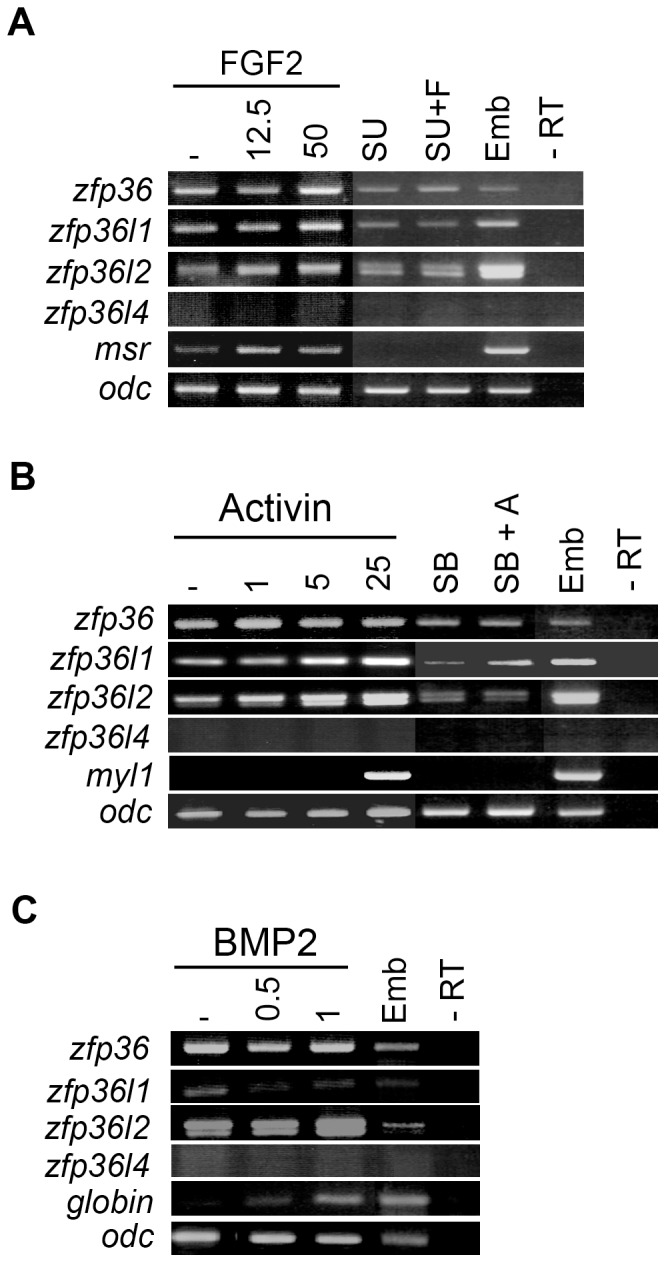
The different *zfp36* genes respond differently to growth factors treatment in animal cap explants. (A) RT-PCR analysis of *zfp36* gene expression in animal cap explants treated with 12.5 or 50 ng/ml of FGF2 or with the FGF inhibitor in the absence (SU) or in the presence of 50 ng/ml FGF2 (SU+F). (B) RT-PCR analysis of *zfp36* gene expression in animal cap explants treated with 1, 5 or 25 ng/ml of activin or with the activin inhibitor in the absence (SB) or presence of 25 ng/ml activin (SB+A). (C) RT-PCR analysis of *zfp36* genes in on animal cap explants from embryos injected with 0.5 ng or 1 ng of BMP2 mRNA. Stage 20 embryo (Emb) or uninjected embryo or untreated animal caps (-) were assayed by RT-PCR for the expression of control genes *msr*, *myl1* and *globin. Odc* was used as control of loading and a reaction was performed in the absence of reverse transcriptase to check for genomic DNA contamination (-RT).

The mammalian ortholog of *Xenopus zfp36* is a primary response gene that is rapidly and transiently induced in fibroblasts when treated with serum and several mitogen factors such as FGF, PDGF or insulin [3], [5]. To determine whether the expression of *zfp36* gene family members was modulated by growth factors, we used the animal cap assay and tested the effects of FGF, activin and BMP that are major signalling pathways acting in the early embryo [29]. FGF2 treatment of animal caps resulted in a dose-dependent increase in expression of *zfp36, zfp36l1* and *zfp36l2* (Fig. 4A). This increase is specific and is not observed in the presence of SU5402 (SU), an inhibitor of the FGF signalling pathway (Fig. 4A). Activin treatment stimulated the expression of *zfp36l1* and *zfp36l2* but had no effect on *zfp36* expression (Fig. 4B). The effect of activin on *zfp36l1* and *zfp36l2* expression is not observed in the presence of SB431542 (SB), an inhibitor of activin signalling pathway (Fig. 4B). Animal caps derived from BMP2 mRNA-injected embryos showed a decrease in *zfp36l1* expression and an increase in *zfp36l2* expression, but no difference in *zfp36* expression whatever the amount of injected mRNA (Fig. 4C). In all those experiments, *zfp36l4* was never expressed and proved totally unresponsive to growth factor treatments. These data indicate that the different *zfp36* genes respond differently to the major signalling pathways that are active in the early embryo.

### Gain-of-function of *zfp36* genes causes somites defects

**Figure 5:**
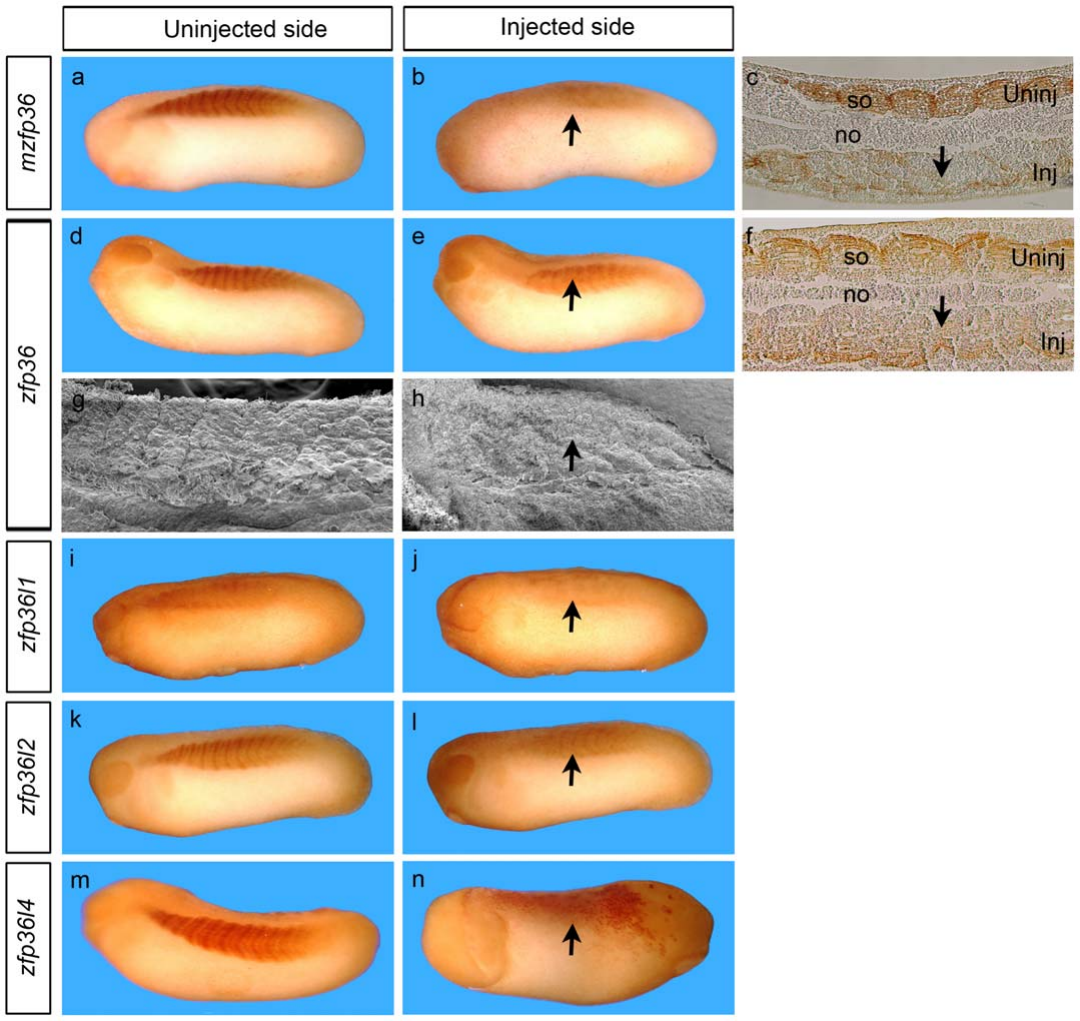
*Zfp36* mRNA overexpression induces somites segmentation defects. 250 pg of mouse *zfp36* mRNA (a, b) or *Xenopus zfp36* (d, e), *zfp36l1* (I, j), *zfp36l2* (k, l) *or zfp36l4* (m, n) mRNA were injected into one blastomere of two-cell stage embryos and developing embryos were fixed at stage 28 before immunhistochemistry analysis with the somite specific marker 12/101. Embryos were embedded in paraffin then sectioned longitudinally (c, f) or treated for scanning electronic microscopy (g, h). The arrows mark the alteration of segmentation on the injected side (Inj) by comparison with the uninjected side (Uninj). no, notochord; so, somite.

As a first step towards understanding the functions of *zfp36* in the early embryo, we expressed the mouse or the amphibian proteins through the injection of their corresponding mRNA. In preliminary experiments, different amounts (from 50 pg to 1 ng) of *zfp36* mRNA were injected into one blastomere of two-cell stage embryos. Injected embryos developed a typical curved axis phenotype, with severity increasing at higher dose. Over 80% (n = 115) of the embryos injected with 250 pg exhibited this highly penetrant phenotype, suggesting a trunk elongation alteration that could be related to alteration in somites (data not shown). To further evaluate somite development, injected embryos were analyzed at tailbud stage by immunhistochemistry with the somite specific marker 12/101. Embryos injected with mouse or amphibian *zfp36* mRNAs showed a slight decrease in 12/101 staining on the injected side compared to the control uninjected side in about 80% of embryos (n = 55) (Fig. 5a, b, d, e). Moreover, the injected side did not show the typical blocks of regularly spaced somites but instead a uniform labeling (Fig. 5b, e). This is more obvious in histological sections (Fig. 5c, f). The ultrastructural defects of somites were confirmed by electron microsocopy analysis (Fig. 5g, h). The injection of *zfp36l1, zfp36l2* or *zfp36l4* mRNAs resulted in the same phenotype (Fig. 5i–n). In these experiments, notochord was not apparently altered (Fig. 5c or 5f) a finding confirmed by immunohistochemistry with the specific antibody MZ15 [30] (data not shown).

**Figure 6:**
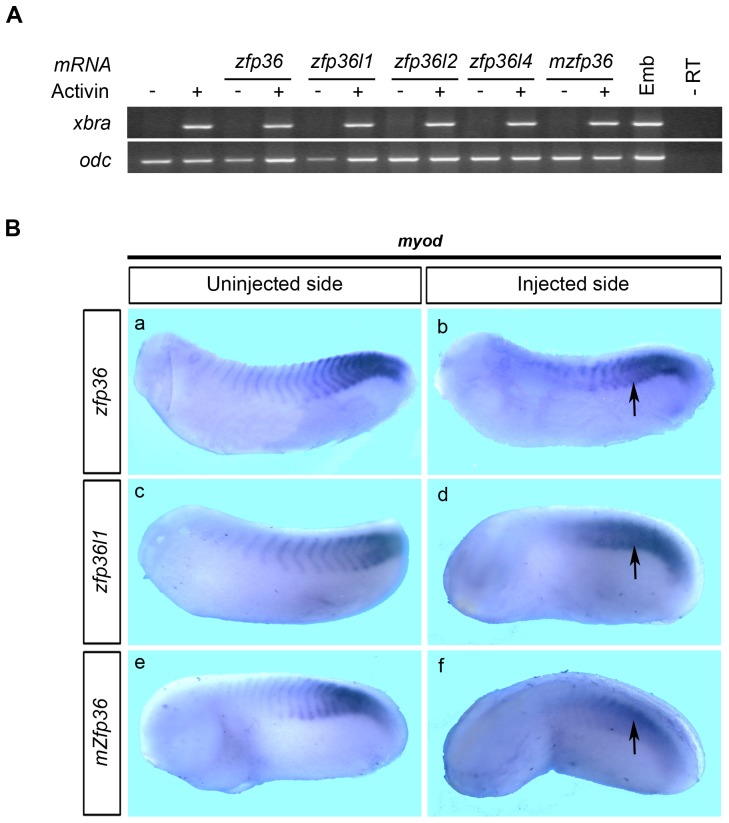
*Zfp36* mRNA overexpression does not prevent mesoderm induction nor myogenic factor expression. (A) Two-cell stage embryos were injected with 250 pg of the different *Xenopus zfp36* mRNAs or mouse *zfp36* mRNA (*mzfp36*) and animal caps were explanted at stage 8.5–9 then treated with 10 ng/ml of activin before analysis by RT-PCR for *xbra* expression when control embryos reached stage 12. Stage 12 embryo (Emb) or untreated animal caps (-) were assayed by RT-PCR in parallel. *Odc* was used as control of loading and a reaction was performed in the absence of reverse transcriptase to check for genomic DNA contamination (-RT). (B) 250 pg of *Xenopus zfp36* (a, b) and *zfp36l1* (c, d) or mouse *Zfp36* (*mZfp36*, e, f) mRNAs were injected in one blastomere of two-cell stage embryos and developing embryos were fixed at stage 28 and analyzed by *in situ* hybridization for *myod* expression.

The reduction in somite staining with 12/101 antibody observed in some cases could result from an impairment in mesoderm induction and/or myogenic differentiation. In order to establish whether *zfp36* mRNA overexpression could affect mesoderm induction, animal cap explants derived from microinjected embryos were treated with activin and assayed for the expression of the pan-mesodermal marker *brachyury* (*xbra*). Animal cap cells derived from microinjected embryos showed no notable change in the expression of *xbra*, indicating that mesoderm induction is not affected by *zfp36* gene expression levels (Fig. 6A). We observed identical results when *zfp36l1, zfp36l2, zfp36l4* or mouse *zfp36* mRNAs are injected (Fig. 6A). To evaluate the effect of *zfp36* overexpression on myogenic differentiation, injected embryos were analysed by *in situ* hybridization for the expression of the myogenic regulatory gene *myod*. Embryos injected with *Xenopus zfp36* mRNA showed *a* more diffuse *myod* expression on the injected side with an altered segmentation pattern compared to the uninjected side (Fig. 6B, a, b). The same results were observed when *zfp36l1, zfp36l2, zfp36l4* or mouse *zfp36* mRNAs were injected (Fig. 6B, c–f and data not shown). Together these results indicate *zfp36* gain-of-function does not affect mesoderm induction or myogenic differentiation but rather impairs somite segmentation.

### Alteration of *esr5* and *hairy2*a expression pattern

**Figure 7:**
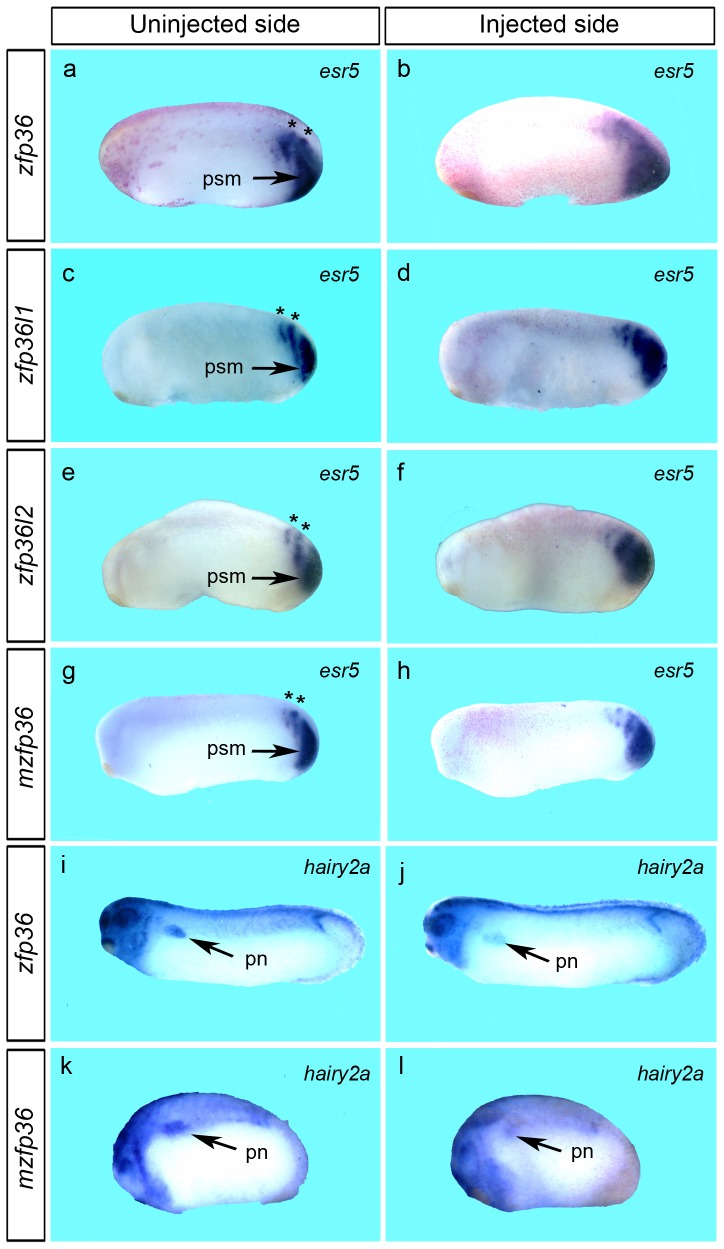
*Zfp36* mRNA overexpression alters the expression of notch signalling pathway members *esr5* and *hairy2a*. 250 pg of *Xenopus zfp36* (a, b, i, j), *zfp36l1* (c, d), *zfp36l2* (e, f) or mouse *zfp36* (*mzfp36*, g, h, k, l) mRNAs were injected into one blastomere of two-cell stage embryos and developing embryos were fixed at stage 25 (a–h and k, l) or stage 28 (i, j) and analyzed by *in situ* hybridization for *esr5* and *hairy2a* expression. Arrows in a, c, e and g mark the presomitic mesoderm (psm) and the pronephros region (pn) in i–l respectively. Stars in a, c, e and g mark the first two somitomeres.

The Notch signaling pathway is central to somitogenesis by controlling somite segmentation through downstream components like *esr5* and *hairy2a* [31], [32]. In order to know whether members of the Notch pathway were affected in gain-of-function experiments, we analysed the expression of *esr5* and *hairy2a* in embryos injected with *zfp36* mRNA. *Esr5* is expressed in a posterior tailbud domain of the embryo marking the presomitic mesoderm, and also in the anterior part of the first two somitomeres, forming two chevrons (Fig. 7a) [32]. Unilateral injection of *zfp36* mRNA resulted in embryos showing no clear demarcation between the two chevrons and in some cases only one chevron was detected (72% n = 45) (Fig. 7b). The same phenotype was observed with embryos injected with *zfp36l1, zfp36l2* or mouse *zfp36* mRNAs (Fig. 7c–h). In the tailbud embryo, *hairy2a* expression is normally found in the presomitic mesoderm as a chevron-shaped stripe and also in the pronephros anlagen (Fig. 7i, k). Embryos injected with either *Xenopus* or mouse *zfp36* mRNA showed a reduced expression of *hairy2a* in both the somites and pronephros anlagen (78% n = 50) (Fig. 7j, l). We conclude from these data that overexpression of zfp36 can indeed affect the Notch signaling pathway.

### Pronephros defects

**Figure 8:**
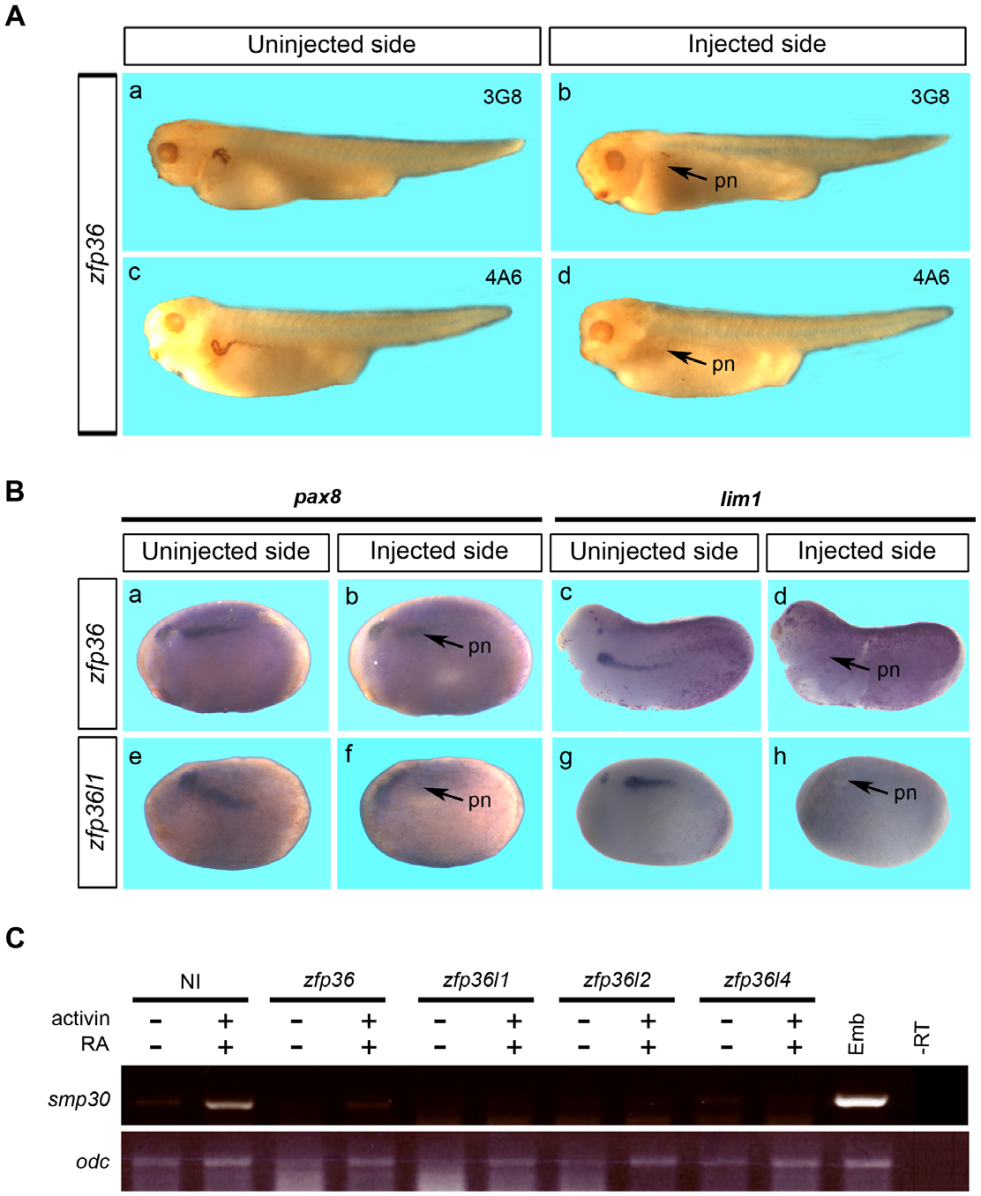
*Zfp36* mRNA overexpression alters the formation of pronephros and affects pronephric marker genes expression. (A) 250 pg of *Xenopus zfp36* mRNA were injected into one ventral blastomere of 8-cell stage embryos and developing embryos were fixed at stage 39 before immunhistochemistry analysis with the pronephros specific markers 3G8 and 4A6. Arrows in b and d mark the pronephros (pn) alteration on the injected side. (B) 250 pg of *Xenopus zfp36* (a–d) or *zfp36l1* (e–h) mRNA was injected into one ventral blastomere of 8-cell stage embryos and developing embryos were fixed at stage 22 (a, b and e–h) or stage 26 (c, d) before *in situ* hybridization analysis for *pax8* or *lim1* expression. Arrows in b, d, f and h mark the pronephros (pn) alteration on the injected side. (C) Two-cell stage embryos were injected or not (NI) with 250 pg of the different *Xenopus zfp36* mRNAs. Animal caps were explanted at stage 8.5–9 and treated with activin plus retinoic acid (RA) before analysis by RT-PCR for *smp30* expression when control embryos reached stage 35. Stage 35 embryo (Emb) or untreated animal caps (-) were assayed by RT-PCR in parallel. *Odc* was used as control of loading and a reaction was performed in the absence of reverse transcriptase to check for genomic DNA contamination (-RT).

Previous studies have shown that overexpression of either *zfp36l1* or *zfp36l2* mRNA in *Xenopus* embyo induces pronephros, abnormalities mainly affecting morphological development of pronephric tubules [25], [27]. Because *zfp36*, like *zfp36l1* and *zfp36l2*, is expressed in pronephros and can alter *hairy2a* expression in the pronephros anlagen (see Fig. 7j), we analysed whether its overexpression could affect pronephros development. Embryos injected at the 8-cell stage in ventral balstomere with *zfp36* mRNA were allowed to develop until tadpole stage and then whole-mount immunostained with the monoclonal antibodies 3G8 and 4A6, markers of pronephric proximal tubule and pronephric duct respectively [33]. Unilateral injection of *zfp36* mRNA resulted in a reduction of tubule and duct staining in embryos (85% n = 35) (Fig. 8A, a–d). Microinjection of *zfp36l1* or *zfp36l2* mRNA gave reproducibly an alteration of pronephros development, thus confirming published data (Data not shown) [25], [27]. In these experiments, we consistently found that the somites were not altered when analysed by immunohistochemistry with the somite specific marker 12/101 antibody, confirming a direct effect of zfp36 overexpresssion on pronephros development (data no shown).

To further investigate the effect of *zfp36* overexpression on pronephros development, we analyzed the expression of *pax8* and *lim1*, key players in pronephros development [34], [35]. Injection of *zfp36* or *zfp36l1* mRNAs resulted in a marked reduced expression of *pax8* and *lim1* (80%, n = 55) (Fig. 8B, a–h). Embryos injected with *zfp36l2* mRNA showed a reduction of *pax8* expression but no change in *lim1* expression (data not shown) as previously reported [25].

To confirm a role of *zfp36* in pronephros development, we used the pluripotent animal cap cells model where pronephros tissue can be induced by treatment with activin and retinoic acid [35], [36], [37]. Animal caps derived from embryos injected with the different *zfp36* mRNAs were assayed for the expression of *SMP30*, a specific marker of pronephric tubule that is induced under conditions where pronephros is formed [38]. A strong inhibition of *SMP30* expression is observed in animal caps injected with *zfp36* mRNAs when compared to non injected control caps (Fig. 8C). We conclude that overexpression of zfp36 affects pronephros development.

### Loss-of-function *of* zfp36 affects pronephros morphogenesis

**Figure 9:**
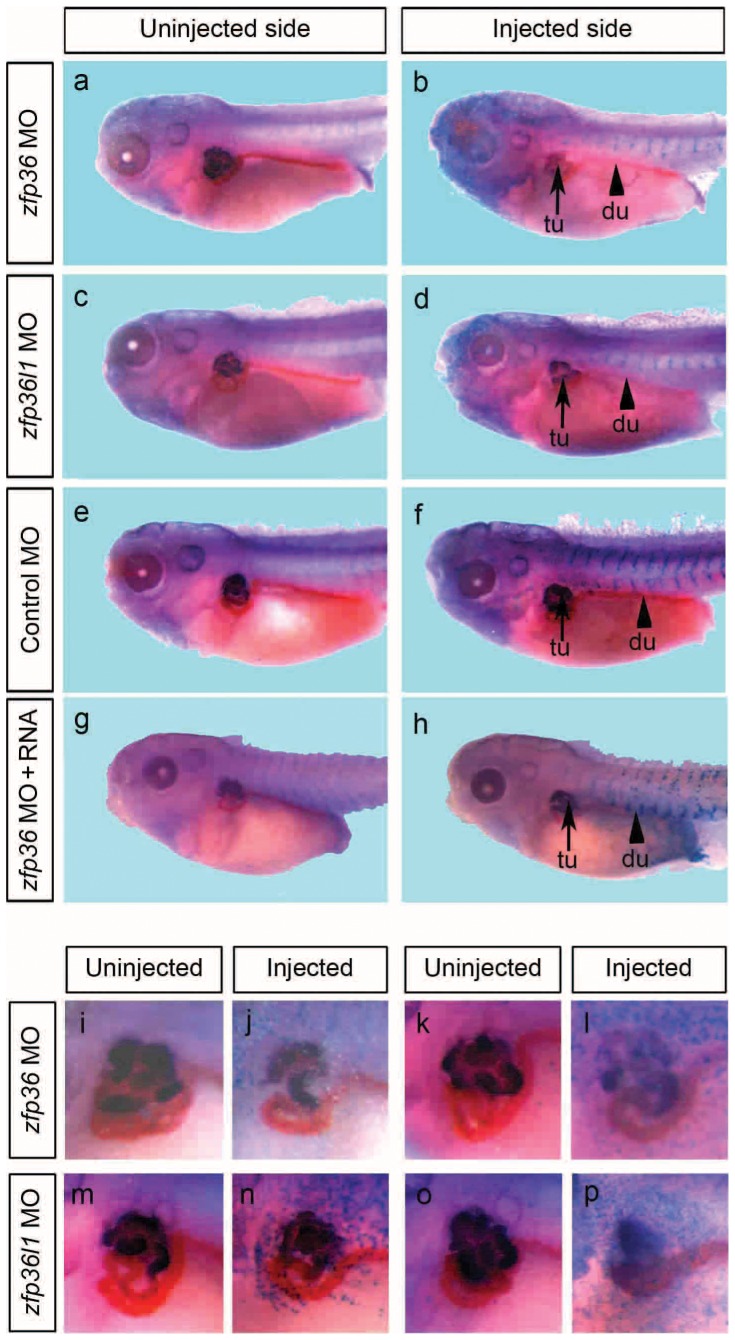
*Zfp36* and *zfp36l1* morpholino knock down induces pronephros alterations. 20 ng of morpholinos directed against *zfp36* (a, b) or *zfp36l1* (c, d) mRNAs or control morpholinos (e, f) were injected into one ventral blastomere of 8-cell stage embryos with 250 pg of *lacZ* mRNA. In rescue experiments, 100–200 pg of mouse *zfp36* mRNA were co-injected with 20 ng of MO *zfp36* (g, h). Developing embryos were fixed at stage 40 before *lacZ* staining and immunohistochemistry analysis to reveal the expression of pronephros specific markers, 3G8 and 4A6. Arrows and arrowheads in b, d, f and h, mark the pronephros proximal tubule (tu) and duct (du) respectively on injected sides of the embryos. I–p, Close up views of anterior region showing uninjected or injected sides of representative phenotypes for *zfp36* morphants (i–l) and *zfp36l1* morphants (m–p).

*Zfp36l1* and *zfp36l2* have been previously shown to affect kidney development either in gain-of-function (for *zfp36l1* and *zfp36l2*) or loss-of-function experiments (for *zfp36l2*) [25], [27]. *Zfp36* which is also expressed in pronephros (See Fig. 3c) may be potentially involved in its development. We tested this hypothesis by loss-of-function analysis using a morpholino antisense (MO) knockdown assay. We also tested similarly the effects of *zfp36l1* knockdown because only gain-of-function has so far been described for this gene [27]. We designed two morpholinos to interfere specifically with the translation of each mRNA. The efficiency of MOs to inhibit translation of their respective mRNAs was established in an *in vitro* reticulocyte lysate system and *in vivo* in the embryo (Fig. S2 and data not shown). MOs were injected in 8-cell stage embryos to target pronephros anlagen and the development of the pronephros was evaluated in tadpole embryos by immunohistochemistry with 3G8 and 4A6 antibodies. Knockdown of either *zfp36* and *zfp36l1* resulted in a similar phenotype characterized by alteration of pronephric tubule morphology, while injection of control MO had no effect (Fig. 9a–f). The phenotype of *zfp36* morphants ranged from a mild to strong size reduction of the tubule, with a defective coiling of the duct (84%, n = 56) (Fig. 9b). *Zfp36l1* morphants also showed alteration of tubule size but, unlike *zfp36* morphants, they also displayed alteration of the distal portion of the duct which is missing (80%, n = 66) (Fig. 9d). Magnified views of the injected side of selected morphant embryos clearly show alteration of tubule morphogenesis (Fig. 9i–p). Morphant embryos also developed edemas, visible from stage 42, a possible consequence of impaired pronephric function [39] (data not shown). The pronephros defects in *zfp36* morphants can be rescued by co-injection of 100 to 200 pg of mouse *zfp36* mRNA (70%, n = 36) indicating that the knockdown effect was specific (Fig. 9g, h).

**Figure 10:**
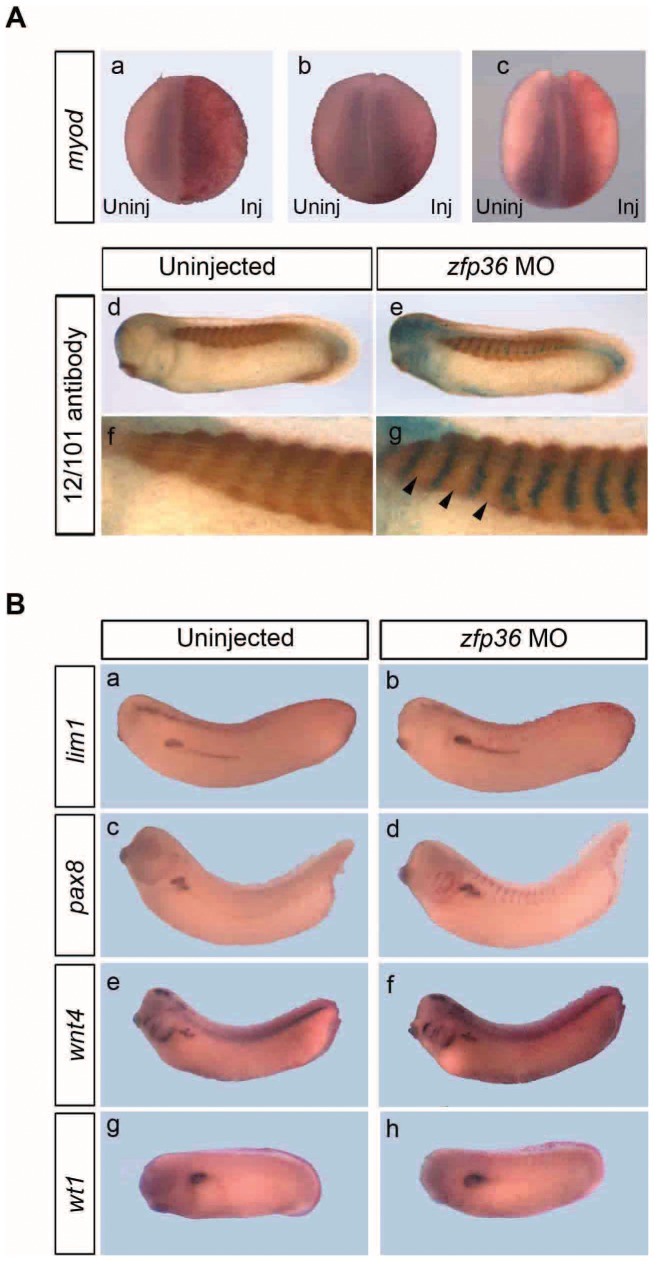
*Zfp36* depletion does not affect somitogenesis nor early pronephros specification. (**A**) 20 ng of *zfp36* morpholinos (MO) were injected into one dorsal blastomere of 4-cell stage embryos with 250 pg of *lacZ* mRNA. Embryos were fixed at stage 15 for the detection of *myod* by in situ hybridization (a–c) or at stage 28 for immunohistochemistry with 12/101 antibody (d–g). a, b and c are representative phenotypes and f and g are close up views of d and e respectively. Arrowheads indicate regularly segmented somites on the injected side. (B) 20 ng of *zfp36* morpholinos (MO) were injected into one ventral blastomere of 8-cell stage embryos with 250 pg of *lacZ* mRNA. Embryos were fixed at stage 29/30 (a, b, e, f), 33/34 (c, d) or 27 (g, h) and analysed for the expression of *lim1, pax8, wnt4* and *wt1* by in situ hybridization.

It is known that signals from anterior somites are involved in pronephros formation. Therefore, we assessed whether MO depletion of zfp36 could affect the formation of paraxial mesoderm formation, thus contributing indirectly to pronephros defects. 4-cell stage embryos were injected dorsally with *zfp36* MO and then analysed at stage 15 for *myod* expression by in situ hybridization and at stage 30 by immunohistochemistry with an antibody (12/101) that specifically labels the somites. *Myod* expression was unchanged in morphants embryos (n = 18) and the labeling of somites with 12/101 antibody was not affected, showing the typical regularly spaced chevron-like structures (n = 23) (Fig. 10A). Taken together, we conclude that targeting *zfp36* morpholinos to mesoderm does not alter somite formation and thus the depletion of zfp36 is likely to have a direct effect on pronephros development.

In order to characterize in more detail the phenotype of *zfp36* morphants, we next asked whether pronephros specification was affected by loss of *zfp36* expression. Several genes have been shown to be implicated in early specification and development of the pronephros, including *lim1, pax8, wnt4* or *wt1* [34], [40], [41], [42], [43]. We did not observe any change in the expression of *lim1* (n = 31)e, *pax8* (n = 33), *wnt4* (n = 25) or *wt1* (n = 22) in *zfp36* morphants (Fig. 10B) indicating that zfp36 is not required for early specification of kidney.

Notch signaling has been shown to play an important role during pronephros development [40], [44], [45]. As components of this pathway are potential zfp36 targets, we have tested whether hairy2a expression, a downstream effector of the pathway, was affected in morphant embryos. Embryos injected with zfp36 MO were cultured until stage 33/34 and analysed for *hairy2a* expression. We did not observe any change in the expression of *hairy2a* (n = 25) indicating that Notch signaling pathway was not affected by zfp36 depletion.

The pronephric defects in *zfp36* morphants could be explained by a decrease of proliferation or the apoptotic elimination of pronephric cells. To evaluate these possibilities, embryos were injected at the 8-cell stage in prospective pronephric territory with zfp36 MO and then analysed for proliferation by immunohistochemistry with an antibody recognizing phosphorylated-histone H3, and for apoptosis by TUNEL. No significant alteration of proliferation (n = 25) or apoptosis (n = 32) was observed on the injected side compared with the uninjected side (Fig. S3). We conclude that the pronephric defects observed in *zfp36* morphants are not related to changes in proliferation or apoptosis.

## Discussion

The present work extends our knowledge on *zfp36* genes and provides a functional comparison between the vertebrate members of this family using *Xenopus* as an experimental model. *Zfp36* genes have evolved from a single gene present in basal metazoans. Indeed, in our survey of extant animals genomes containing a Tandem Zinc Finger domain identical to human *ZFP36*, we have identified a single gene present in the genome of basal phylum such as porifera (Amphimedon) and cnidaria (Nematostella). There is also a single *zfp36* gene in protosotomes (*Drosophila, C.elegans* or mollusc) and in basal deusterostomes such as echinoderm (sea urchin) or urochordates (ciona). In contrast, there are three to four *zfp36* genes in vertebrates, depending on the species, in agreement with the hypothesis that two rounds of duplication have occurred between the origin of chordates and the origin of jawed vertebrates [28]. Of the four *zfp36* genes present in rodent and amphibian, three of them, ie *zfp36, zfp36l1* and *zfp36l2*, are true orthologs while the fourth one, *zfp36l3* in rodent and *zfp36l4* in amphibian has probably arisen through retrotransposition of a processed cDNA. When considering gene structure, one striking finding is that there is strong conservation between the unique *zfp36* gene found in sponges and the deuterostome *zfp36* orthologs, where the unique intron is found at the same position and in the same phase. Moreover, in all genes analyzed, the TZF domain is constantly found in the second exon. This conservation, that has been maintained over more than 800 My of evolution, is not found in protostomes genomes of *Drosophila melanogaster* and *C. elegans* where *zfp36* genes show an increased number of introns. This suggests that the common ancestor of bilaterian *zfp36* had a simple gene structure with two exons and that the variation in gene number (observed for example in *Drosophila melanogaster* or *C.elegans*) reflects secondary lineage specific gain of introns [46].

We have used the *Xenopus* embryo to evaluate whether different *zfp36* genes could have distinct functions during early development. Our work demonstrates that FGF, activin and BMP, some of the major signaling pathways during early Xenopus development, are implicated at some level in the differential regulation of *zfp36* in the embryo. Zfp36 are RNA binding proteins that recognize AU-rich element within 3′UTR of mRNAs leading to their deadenylation and accelerated degradation [7]. We may therefore hypothesize that their function is closely related to their temporal and spatial expression. *zfp36l4* is maternally expressed and not detected after midblastula transition and therefore has probably no function in early development and organogenesis. Indeed, it has been shown to be required for meiosis progression [26]. *Zfp36l1* and *zfp36l2* expression patterns are closely similar in the embryo [25], [27]. Although *zfp36* is expressed in pronephros like *zfp36l1* and *zfp36l2*, it differs from the two other, being the sole member of the family expressed in the notochord. Moreover, when considering pronephros development, *zfp36* expression is delayed when compared to the two other genes. This expression pattern could reflect distinct regulatory elements that are shared by *zfp36l1* and *zfp36l2* and their common origin through genome duplication during the course of evolution while *zfp36* regulatory regions would have evolved independently.

When overexpressed in the embryo, each of the four zfp36 proteins induces somite segmentation defects and pronephros alteration without affecting myogenesis or somite formation. In agreement with the indispensable requirement of Notch signalling for somite segmentation, we have found that the expression of *esr5 and hairy2a* mRNAs, two members of the signalling pathway, is altered. Those mRNAs possess an ARE in their 3′UTR and therefore can be targeted for degradation by zfp36 proteins (data not shown). A survey of mRNA possessing ARE elements in their 3′UTR indicates that several members of the Notch signaling pathway, such as *Notch* itself, *Delta* or *esr9* mRNAs, could be direct targets of zfp36 proteins (data not shown). The direct involvement of ZFP36 in Notch signalling has been recently described in human thymocyte development where a direct interaction between ZFP36L1 or ZFP36L2 and an ARE present in the 3′UTR of *Notch1* mRNA has been observed [18]. A striking finding is that the three predicted binding sites for ZFP36 in the mammalian 3′UTR *Notch1* mRNA are totally conserved in the *Xenopus* 3′UTR *notch* mRNA (data not shown). Among the three binding sites, there is a nonamer sequence UUAUUUAUU that has been described as the optimum binding site for all zfp36 family members [11], [47], [48]. Interestingly, this sequence is also found in the *Xenopus hairy2a* 3′UTR reinforcing the idea that several members of Notch pathway signalling can be targeted by zfp36 proteins. Surprisingly, this nonamer sequence is also found in the 3′UTR region in *WT1* (Wilms tumor suppressor) mRNA that encodes a protein that has been shown to be involved in nephron defects in vertebrates [49]. However, we cannot exclude the possibility that alteration of somite segmentation may also be indirect and a consequence of the targeting by zfp36 of mRNAs that themselves my regulate somite segmentation. For instance, one such target could be *EDEN BP* mRNA whose downregulation disrupts *esr5* expression pattern in a way similar to zfp36 [50]. Several other mRNA encoding RNA binding proteins have also been shown to regulate somitogenesis and can be potentially targeted by zfp36 [51], [52].

Notch activation is essential for pronephros development both in *Xenopus* and in mouse, acting on proximal tubule and glomus formation [40], [44], [53], [54], [55]. We can hypothesize, from our gain-of-function experiments, that zfp36 acts through the targeting of Notch signalling pathway elements, like hairy2a or esr5, to alter pronephros formation. Zfp36 can also act at an early step in pronephros development and target important regulators such as *pax8* or *lim1* mRNA whose expression is significantly decreased in zfp36 overexpressing embryos. These data confirm previous observations made on *zfp36l1* and *zfp36l2* [25], [27]. During this work, we have constantly found that the mouse Zfp36 protein induces the same embryonic defects than the amphibian proteins, suggesting a functional conservation between vertebrate proteins. We have a limited knowledge of mRNA targets of zfp36 family members and whether individual members of the family target distinct, overlapping, or identical targets to other family members. Our gain-of-function experiments provide evidence that the distinct zfp36 proteins give the same somite segmentation and pronephros defects, suggesting that the different zfp36 proteins may have the same mRNAs targets.

Loss-of-function using morpholino antisense oligonucleotides interfering with *zfp36* translation shows an extremely consistent phenotype characterized by a dramatic alteration of pronephros development leading to tubule size reduction and coiling defects. Those defects, that are not related to somites alteration can be rescued by the mouse zfp36 protein, indicating that they are specific but also that there is a strong functional conservation between the amphibian and the mammalian proteins. The expression of genes involved at early stages of pronephros formation such like *lim1, pax8, wnt4* or *wt1* is not affected in zfp36 morphants embryos, nor is apoptosis or proliferation. Notch pathway, at least its downstream effector *hairy2a*, seems not also affected by zfp36 depletion. We conclude from our experiments that *zfp36* expression is not essential at early step of the pronephros specification, but is critically required at a later step of its organogenesis. We have shown for the first time that this is also the case for *zfp36l1*. Our data extend previous work that showed that *zfp36l2* knock down impaired pronephros development [25]. Therefore, from our report and from published work, all zfp36 gene family members that are zygotically expressed in the early Xenopus embryo are necessary for a correct pronephros development. One striking issue that emerges from morphant analysis is the complete lack of redundancy between those genes while overexpression of all members induce the same phenotype. In over expression studies, zfp36 proteins are expressed in all regions of the embryo and therefore they can target the same mRNA as long as they possess ARE in their 3-UTR. We have shown this can be the case for several Notch signaling members. In the case of knockdown, the lack of redundancy between zfp36 family members could be explained by the distinct spatial and temporal expression of each gene. *Zfp36l1* and *zfp36l2* are expressed in pronephros anlagen but not in a totally overlapping pattern, while *zfp36* expression, in contrast to the two others, is temporally delayed and restricted to tubule expression. This suggests that, although they can target the same mRNAs, the different zfp36 proteins are not acting at the same development stage or in the same cells. However we cannot exclude that, even when co expressed in the same cells, the individual zfp36 proteins interact with distinct partners that are necessary for their function. One puzzling observation from our work is that both gain-of-function and loss-of-function strategies affect pronephros development in a similar way. We suggest that a fine balance between the different zfp36 proteins level is required for normal development. Nevertheless, gain-of-function and loss-of-function studies differ in the way they affect early steps in pronephros development. Whereas *lim1* and *pax8* expression is decreased in embryos overexpressing zfp36 proteins, their expression, like those of *wnt4*, or *wt1* (two others major actors in pronephros development) is unchanged in zfp36 morphants embryos. We hypothesize there exist additional targets to be discovered, whose expression is finely tuned by zfp36 proteins at late stages of pronephros morphogenesis.

Although zfp36 proteins are predominantly located in the cytoplasm, an unexpected partner that has been described in the case of zfp36l1 is the transcription factor HNF1β which, when mutated, is responsible for kidney congenital defects [27]. In a search for mutations in the open reading frame of human *ZFP36L1* in patients with renal anomalies none were found [27]. Because *Zfp36l1* knock down in mouse is embryonic lethal, we may hypothesize that mutations in the human protein might also be deleterious for development. Since our results indicate that zfp36 and zfp36l1 have similar effects on pronephros development, it is conceivable that some human renal anomalies might be related to zfp36 mutations, thus opening new interesting investigations. Together our studies indicate that *zfp36* gene family members have unique function during pronephros development and suggest a model in which they regulate late phase of organogenesis. While zfp36 proteins have previously been reported to be involved in inflammatory disease and cancer, our study establishes an additional critical role during kidney development and morphogenesis. Given the conservation in gene structure and function between the amphibian and mammalian proteins and the conserved mechanisms for pronephros development our studies have uncovered a potential role of *zfp36* gene in human kidney disease that merits further investigation.

## Materials and Methods

### Ethics Statement

This study was carried out in strict accordance with the recommendations in the Guide for the Care and Use of Laboratory Animals of the European Community. The protocol was approved by the “Comité d'éthique en expérimentation de Bordeaux» N° 33011005-A.

### Plasmid constructions

The coding sequences of the four *Xenopus laevis zfp36* genes have been cloned into pCS2 vector by PCR using primers containing restriction sites. *Zfp36* cloning was made from the IMAGE clone 7009009 (Accession BC082435) while *zfp36l1, zfp36l2* and *zfp36l4* sequences were cloned by RT-PCR from embryo RNA. A flag epitope has been added to the C-terminal end of the sequences. Mouse *zfp36* (mZfp36) coding sequence has been cloned by RT-PCR from Embryonic Stem cells RNA in pXT7 vector. The primers for cloning are as follows (cloning sites are underlined):

*zfp36* forward: 5′-GCCCGGATCCGCTTGGTGGGTCAATATGTCCTCTATCCT-3′; 

*zfp36* reverse: 5′-CCGGCGAATTCTCAATCCGACACGGACAACCTGTTAAAG-3′

*zfp36l1* forward: 5′-GGCCGAATTCCTCAAGATGTCTACAGCTTTG-3′

*zfp36l1* reverse: 5′-GGCCTCTGAGACAGGCTTAATCATCAGAGATAG-3′

*zfp36l2* forward: 5′-GGCCGAATTCATGTCTGCGACCCTTTTATCCG-3′

*zfp36l2* reverse: 5′-GGCCTCTAGATGGCTTTAATCATCGCTTATGG-3′

*zfp36l4* forward: 5′-GGCCGAATTCAGCTGGCAATGGAGATATCAAATG-3′

*zfp36l4* reverse: 5′-GGCCTCTAGAGTTAGCCGAGGACAGACAGTAG-3′

*mZfp36* foward: 5′-CTAGTCGACGCCACCATGGATCTCTCTGCCATCTAC-3′

*mZfp36* reverse: 5′-CCGCAGCTGTCACTCAGAGACAGAGATACG-3′.

### RT-PCR analysis

RT-PCR analysis was performed as previously described [56] with the following primer pairs:

*zfp36* 5′-TAAGGATGCCTGCATCTGTC-3′ and 5′-ATCCCTGTTGCTGTAGATGC-3′; *zfp36l1* 5′-GAAGATGCAGGAAGCACCAG-3′ and 5′-CCAAAATGATGGTGGGAAGC-3′; *zfp36l2* 5′-ACATGAGACCATACCACCTC-3′ and 5′-CACATACCTGTCTAAAGCC-3′; *zfp36l4* 5′-TGTGTCTATCACCGGTTCGG-3′ and 5′-TCTTTGGTAACAGAGGCAGG-3′; *odc* 5′-GTCAATGATGGAGTGTATGGATC-3′ and 5′-TCCATTCCGCTCTCCTGACCAC-3′.

*xbra* 5′-TTAAGTGCTGTAATCTCTTCA-3′ and 5′-GCTGGAAGTATGTGAATGGAG-3′

*myl1* 5′-TTTGACAAGGAAGGCAATGG-3′ and 5′-CATTCTGCTGACAGTTCTTG-3′

*smp30* 5′-TTAGACTGGTCTCTGGATCAC-3′ and 5′-CGATAGGTAACTTTACAGTCT-3′

*chordin* 5′-CTCCAATCCAAGACTCCAGC-3′ and 5′-GGAGGAGGAGGAGCTTTGGG-3′

*wnt8* 5′-TGGCAAGAACTTGTCCCAGT-3′ and 5′-TTCTGGAATGCCGTCATCTC-3′

*msr* 5′-ACATCATTGTCAGCCTGCAC-3′ and 5′-AGTCCCTGTTCTGTAATCAG-3′

*globin* 5′-GCTGTCTCACACCATCCAGG-3′ and 5′-TGTACTTGGAGGTGAGGACG-3′.

For an accurate semi quantitative analysis, we have used conditions where the signals obtained are a linear function of the input cDNA as measured by amplification of serial dilutions of the input cDNA (data not shown).

### Microinjection and animal cap assay

*Xenopus laevis* eggs were obtained by injecting adult females with 750U human chorionic gonadotrophin. Staging of embryos was according to Nieuwkoop and Faber tables [57]. For induction assay, animal cap explants were dissected from stage 8.5–9 embryos and treated with different amount of bFGF, activin (R&D Systems) or a mixture of activin plus retinoic acid (SIGMA R2625) and cultured until the control embryos reached the appropriate stage before RT-PCR analysis. The FGF inhibitor SU5402 (SIGMA) and the activin inhibitor SB431542 (SIGMA) were used at 50µM. For microinjection experiments, we used a Nanoject system (Drummond Scientific) and the capped mRNAs were synthesized *in vitro* using Ambion mMessenger mMachine SP6 kit (Austin, TX). We determined in preliminary experiments the effective doses for the microinjection experiments corresponding to 250 pg *zfp36* mRNA and 20 ng to 50 ng of MO. For rescue experiments, 100–200 pg of mouse *Zfp36* mRNA were co-injected with the MOs. 250 pg of *β-galactosidase* mRNA were used as tracer and the injection were performed into one blastomere at either 2-cell stage, 4-cell stage or in a ventral blastomere of 8-cell stage embryos. For animal cap assay, embryos were injected in the animal pole of 2-cell stage embryo into both blastomeres. Animal caps were then dissected at stage 8.5–9 and cultured to the appropriate stage before RNA extraction and RT-PCR analysis. All results shown are representative of at least two independents experiments.

### Whole mount *in situ* hybridization, immunohistochemistry and scanning electronic microscopy

Whole-mount *in situ* hybridization was performed according to standard protocol [58] using antisense digoxigenin-labeled probes and BM purple revelation (Roche). To generate antisense probes, plasmids were linearized and transcribed as follows: *pGEMT-esr5*, NotI/T7; *hairy2a*, SalI/T7 [59]; *lim1*, XhoI/T7; *pGEMT-myod* SpeI/T7 [60]; *pBS-pax8*, SstII/T7; pgem2-wnt4, NheI/T7 [61]; *pSC-B-wt1, EcoRI/T7* [42]; pCS2-zfp36, BamHI/T7; pCS2-zfp36l1, EcoRI/T7, pCS2-zfp36l2, EcoRI/T7; pCS2-zfp36l4, EcoRI/T7. For serial sections, embryos were post fixed in MEMFA for 1 hr at room temperature and embedded in paraffin before cutting 10 µm transverse sections on a microtome. For immunohistochemistry, embryos were collected, fixed in MEMFA with 3.7% formaldehyde, and processed using current protocol [58]. Primary mouse monoclonal antibody 12–101 was used at 1/2 dilution. 3G8 and 4A6 antibodies were a kind gift of Dr. Liz Jones and used at a 1/40 dilution or undiluted respectively [33]. Alkaline phosphatase blue color reaction products were generated using BCIP/NBT and red with Fast Red (Roche). For scanning electron microscopy, embryos were fixed in 100 mM cacodylate buffer and 1.5% glutaraldehyde. After dehydratation in ethanol, critical point was performed in ethanol and liquid nitrogen. Dorsal epithelium was peeled away to show the somites and fractures were performed at various levels.

### TUNEL staining and proliferation assay

The whole-mount TUNEL staining protocol was carried out following the protocol as previously described [62]. The visualization of proliferative cells was performed according to published protocol [63] using a polyclonal anti-phospho Histone H3 (ser 10) (Millipore cat# 06–570, 1∶1,000) antibody and a anti-rabbit horseradish peroxydase – conjugated antibody (Invitrogen G21234, 1∶500).

### Antisense morpholino oligonucleotide

Antisense morpholino oligonucleotides were obtained from Gene Tools LLC. The sequence of the antisense MO was based on the designed parameters recommended by Gene Tools, as follows:

MOzfp36 (5′-ATATCCAGGATAGAGGACATATTGA-3′)

MOzfp36l1 (5′-AGGAGAAATCAATGCTGTAGACATC-3′)

MOzfp36l2 (5′-CGGATAAAAGGGTCGTAGACATTTC-3′)

Standard Control MO (5′-CCTCTTACCTCAGTTACAATTTATA -3′).

### Identification of *zfp36* sequences

Vertebrate zfp36 sequences were retrieved from avalaible databases using the BLAST algorithm and *Xenopus* tandem zing finger domain sequences as query. The different sequences used are from *Strongylocentrotus purpuratus* (XP_782811 and XP_001175665), *Caenorhabditis. elegans* (NM_073525.6), *Ciona intestinalis* (NP_001071879.1), *Drosophila Melanogaster* (NP_511141.2), *Nematostella vectensis* (XP_001624163.1), *Amphimedon queenslandica* (XP_003386486) and *Tribolium castaneum* (NC_007419). Synteny analysis and exon-intron structure of the genes were made using the Ensembl Genome Browser. For some genes we retrieved the genomic region containing *zfp36* sequences and determined the exon intron stucture by comparing genomic and cDNA sequences. Phylogenetic analyses were made using the mega4 program [64].

## Supporting Information

Figure S1 **Phylogenetic tree showing the evolutionary relationship between** ***zfp36, zfp36l1*** **and** ***zfp36l2*** **genes.** The tree was made from the amino acids sequence of the tandem zinc finger domain using mega4 program. *Ae*, Aedes aegypti; *Aq, Amphimedon queenslandica; Ce*, *Caenorhabditis elegans; Ci, Ciona intestinalis; Dm, Drosophila Melanogaster; Hm, Hydra magnipapillata; Hs, Homo sapiens; Io, Ilyanassa obsoleta; Mm, Mus musculus; Nv, Nematostella vectensis; Sc, Saccharomyces cerevisiae; Sp, Strongylocentrotus purpuratus; Sk; Socoglossus kowalevskii; Tc, Tribolium castaneum; Xt, Xenopus tropicalis.*
(TIF)

Figure S2 **Efficacy of** ***zfp36*** **mRNA translation inhibition by morpholinos**. (A) 500 pg of *zfp36* mRNA were *in vitro* translated in reticulocyte lysate and translation products were analyzed by SDS PAGE followed by autoradiography. Lane 0, mock translation without mRNA; lane 1, no *zfp36* MO; lane 2, 50 ng of *zfp36* MO; lane 3, 100 ng of *zfp36* MO; lane 4, 100 ng of Control (Co) MO. (B) 250 pg of *zfp36* mRNA were injected in embryo alone (lane 1) or with 80 ng of *zfp36* MO (lane 2) or 80 ng of control MO (lane 3). Embryos were fixed at stage 12 and protein extracts were analyzed by western blot with an anti flag antibody. The migration of zfp36 protein is indicated by an arrow. Lane 0, uninjected embryo. Non specific signal (ns).
(TIF)

Figure S3 **Impaired pronephros morphogenesis caused by** ***zfp36*** **depletion is largely independent of Notch pathway, proliferation or apoptosis.** 8-cell stage embryos were injected unilaterally with 20 ng of z*fp36* morpholinos together with 250 pg of *lacZ* mRNA tracer and analysed at stage 33/34 for *Hairy2a* expression by whole mount in situ hybridization (a, b), at stage 32 by TUNEL assay (c, d) or at stage 28 by immunohistochemistry with anti-phospho-Histone H3 antibody (Phospho-H3) (e, f).
(TIF)
