# Supplementary figures and images for: Comparative Functional Analysis of ZFP36 Genes during Xenopus Development
Source: PLoS One. 2013 Jan 16;8(1):e54550. doi: 10.1371/journal.pone.0054550 (PMC3546996; doi:10.1371/journal.pone.0054550)

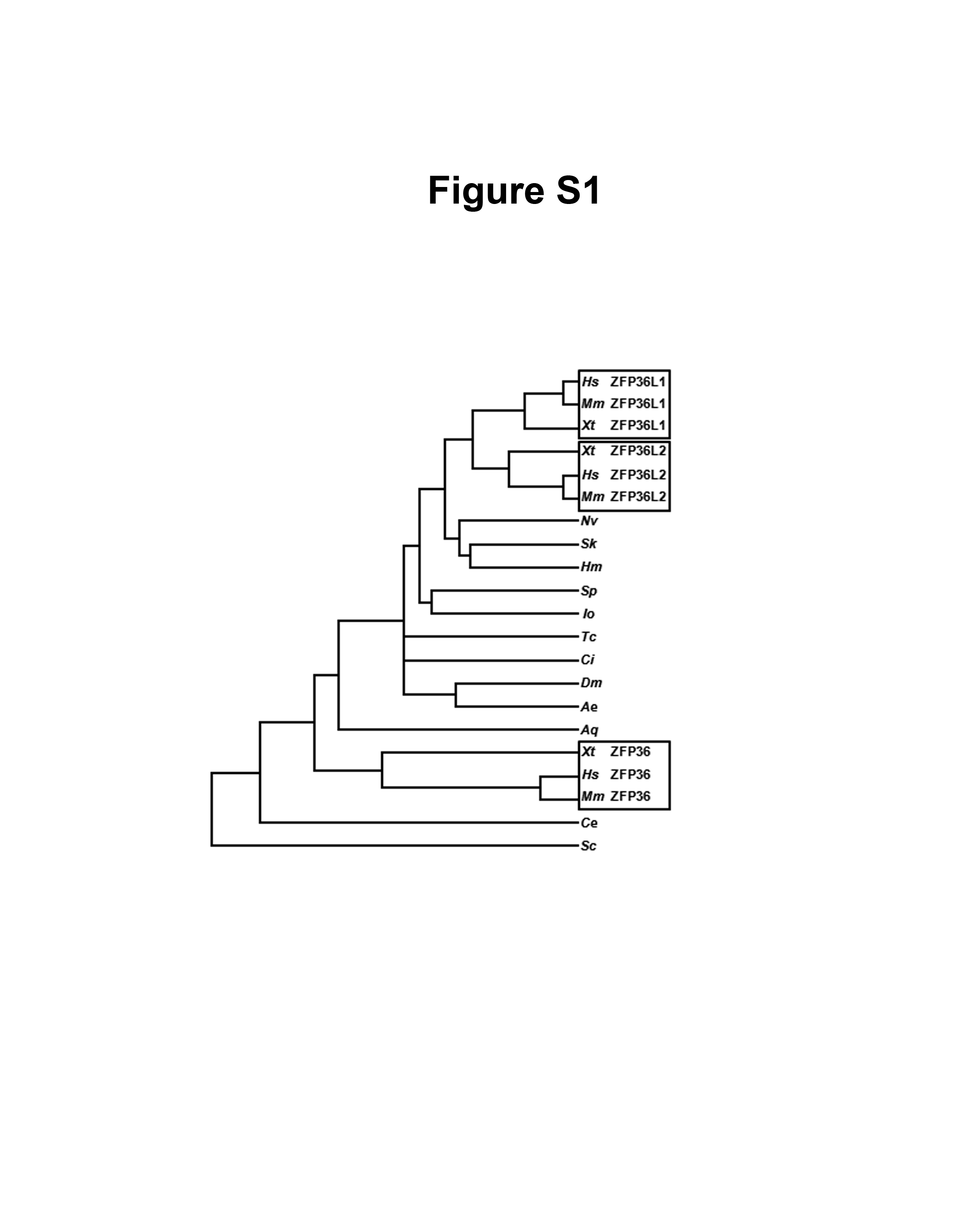

Supplement: Figure S1 — Phylogenetic tree showing the evolutionary relationship between zfp36, zfp36l1 and zfp36l2 genes. The tree was made from the amino acids sequence of the tandem zinc finger domain using mega4 program. Ae, Aedes aegypti; Aq, Amphimedon queenslandica; Ce, Caenorhabditis elegans; Ci, Ciona intestinalis; Dm, Drosophila Melanogaster; Hm, Hydra magnipapillata; Hs, Homo sapiens; Io, Ilyanassa obsoleta; Mm, Mus musculus; Nv, Nematostella vectensis; Sc, Saccharomyces cerevisiae; Sp, Strongylocentrotus purpuratus; Sk; Socoglossus kowalevskii; Tc, Tribolium castaneum; Xt, Xenopus tropicalis. (TIF) [file pone.0054550.s001.tif]

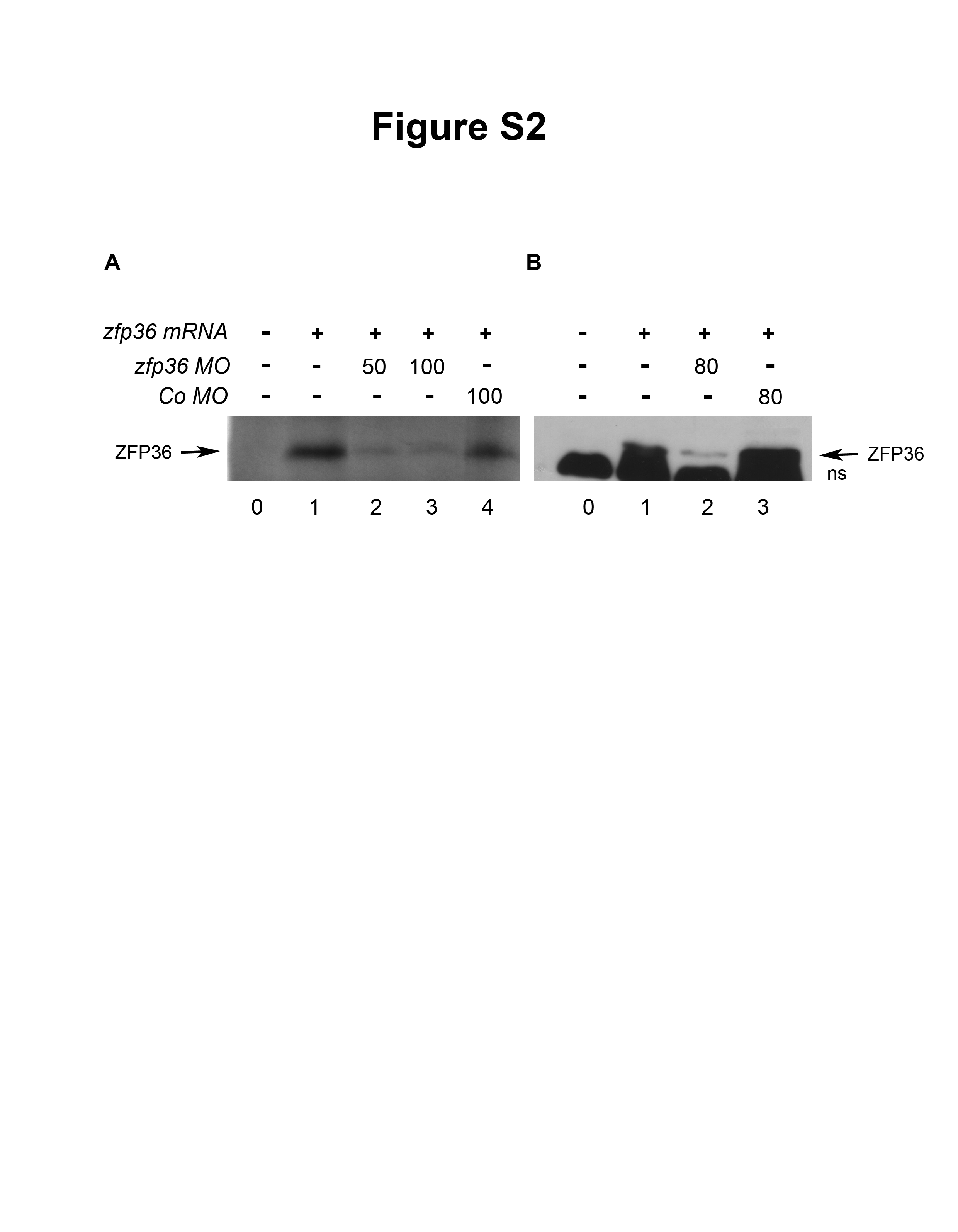

Supplement: Figure S2 — Efficacy of zfp36 mRNA translation inhibition by morpholinos. (A) 500 pg of zfp36 mRNA were in vitro translated in reticulocyte lysate and translation products were analyzed by SDS PAGE followed by autoradiography. Lane 0, mock translation without mRNA; lane 1, no zfp36 MO; lane 2, 50 ng of zfp36 MO; lane 3, 100 ng of zfp36 MO; lane 4, 100 ng of Control (Co) MO. (B) 250 pg of zfp36 mRNA were injected in embryo alone (lane 1) or with 80 ng of zfp36 MO (lane 2) or 80 ng of control MO (lane 3). Embryos were fixed at stage 12 and protein extracts were analyzed by western blot with an anti flag antibody. The migration of zfp36 protein is indicated by an arrow. Lane 0, uninjected embryo. Non specific signal (ns). (TIF) [file pone.0054550.s002.tif]

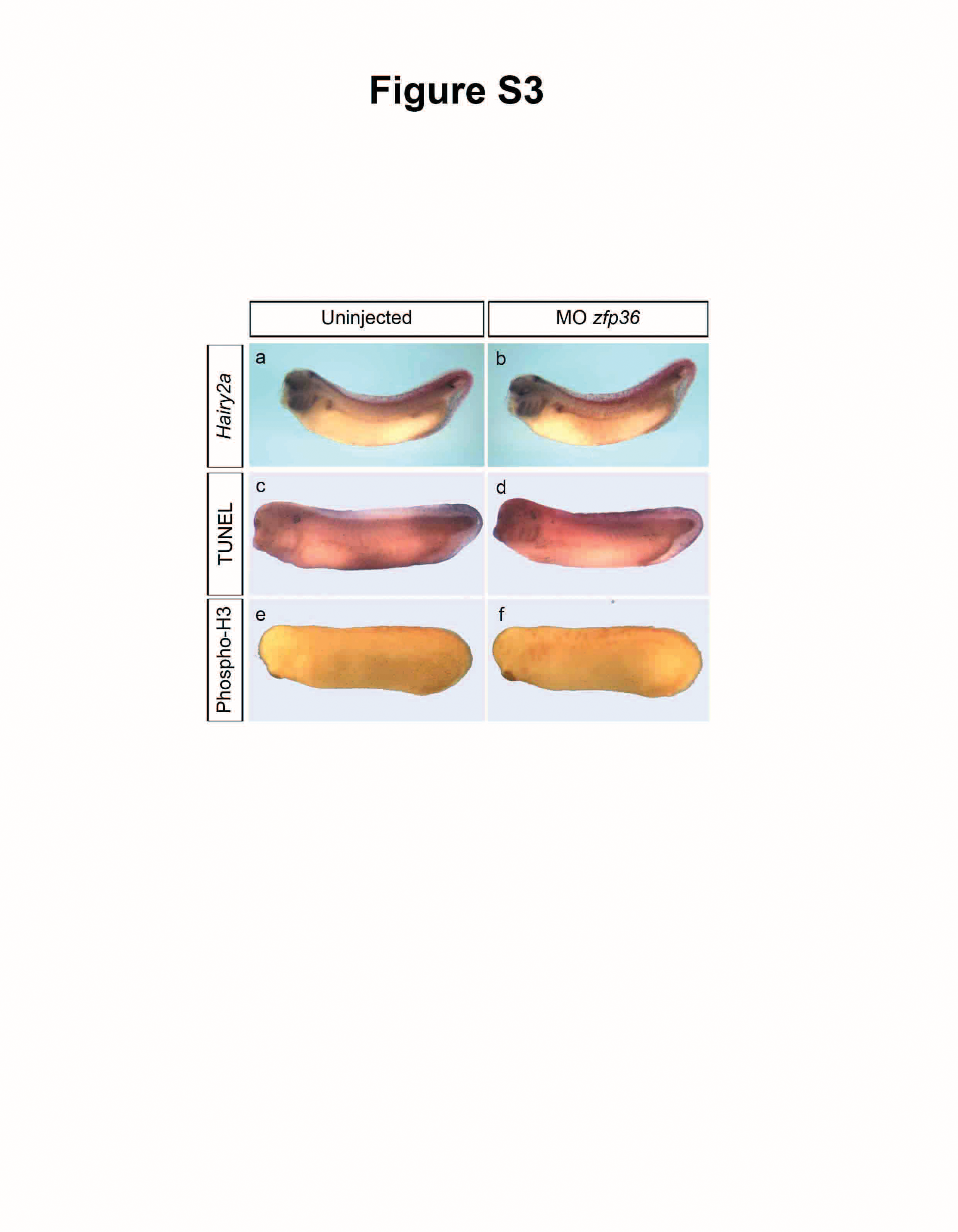

Supplement: Figure S3 — Impaired pronephros morphogenesis caused by zfp36 depletion is largely independent of Notch pathway, proliferation or apoptosis. 8-cell stage embryos were injected unilaterally with 20 ng of zfp36 morpholinos together with 250 pg of lacZ mRNA tracer and analysed at stage 33/34 for Hairy2a expression by whole mount in situ hybridization (a, b), at stage 32 by TUNEL assay (c, d) or at stage 28 by immunohistochemistry with anti-phospho-Histone H3 antibody (Phospho-H3) (e, f). (TIF) [file pone.0054550.s003.tif]
